# Awareness of standard precautions, circumstances of occurrence and management of occupational exposures to body fluids among healthcare workers in a regional level referral hospital (Bertoua, Cameroon)

**DOI:** 10.1186/s12913-024-10855-x

**Published:** 2024-04-03

**Authors:** Innocent Takougang, Fabrice Zobel Lekeumo Cheuyem, Billy Ralph Sanding Ze, Forlemu Fabiola Tsamoh, Hortense Mengong Moneboulou

**Affiliations:** 1 Department of Public Health, Faculty of Medicine and Biomedical Sciences, The University of Yaoundé I, Yaoundé, Cameroon; 2 Department of Odonto-Stomatology & Maxillofacial Surgery, Faculty of Medicine & Biomedical Sciences, The University of Yaoundé I, Yaoundé, Cameroon

**Keywords:** Accidental exposure to blood, Healthcare workers, Hepatitis B vaccination

## Abstract

**Background:**

Healthcare workers (HCW) are exposed to infectious agents within biological materials including blood, tissues, other body fluids and on medical supplies, contaminated surfaces within the care delivery environment. Trends in occupational injuries are influenced by the level of awareness and observance of standard precautions (SP) among HCWs. This study aimed to assess the level of awareness of SP, exposure to body fluids, reporting pattern and management among HCWs in a Referral Hospital.

**Methods:**

The present hospital-based cross-sectional study was carried out from 1st November 2020 to 31st May 2021. The exhaustive sampling method was used and a total of 120 consenting HCWs were invited to participate. A self-administered questionnaire addressed questions related to knowledge, experience, circumstances of exposure, reporting, management of occupational exposure to body fluids, hepatitis B vaccination status. Data were analyzed using R Statistic version 4.3.1. A *p*-value < 0.05 was considered significant.

**Results:**

Out of the 120 participating HCWs, 104 (86.7%) reported at least one accidental exposure to body fluids over the last year. Men (aOR = 4.19; *p* = 0.277) and HCWs aged 35 and over (aOR = 4.11; *p* = 0.114) were more at risk for AEB even though the difference was not statistically significant. Nurses/midwives (aOR = 65.9; *p*-value = 0.0005) and cleaners (aOR = 14.7; *p*-value = 0.0438) faced the highest risk of exposure. Lack of knowledge (79%) and patient agitation (49%) were the most reported reasons for exposure. Half of the participants (53%) reported that they used a personal protective equipment during care. Face mask (59.2%) and gowns (30.8%) were the most commonly used PPE. Most HCWs (62%) did not report AEB. Half of the affected HCWs (50.8%) received a course of post-exposure antiretroviral therapy. Few HCWs (4.2%) were fully immunized against Hepatitis B.

**Conclusions:**

Most HCWs reported an accidental exposure to body fluids over the last year. Midwives and nurses were disproportionally affected socio-professional groups. Two-thirds of the AEB were undeclared. Only half of the participants reported using PPE systematically. Hepatitis B vaccination coverage was low. There is need to strengthen the observance of standard precautions, including preventive vaccination and the systematic reporting and management of AEB.

**Supplementary Information:**

The online version contains supplementary material available at 10.1186/s12913-024-10855-x.

## Background

Occupational exposure to blood and body fluid presents a serious public health concern, especially among healthcare workers (HCW) who are central to achieving the global vision of universal health coverage [1, 2]. HCWs are at risk of exposure to the most virulent communicable disease agents because of their contact with the most affected patients and infective material in their working environment [3, 4]. Infected health workers are likely to transmit the most virulent infections to their vulnerable patients, visitors and communities at high risk for severe disease, complications and death [5]. Infectious materials include body fluids such as blood, urine, saliva, droplets, contaminated tissues, tools, equipment, surfaces and the environment [6]. Accidental exposures occur through needle-stick injuries, cuts by sharp objects or projections of biological fluids [7, 8]. Such exposures increase the risk of transmission of infectious diseases agents such as the Human Immunodeficiency (HIV), Hepatitis B (HBV), and Hepatitis C (HCV) viruses, *Mycobacterium tuberculosis*, COVID-19 and other blood-borne pathogens, especially in Sub-Saharan Africa [7, 8]. Socioprofessional characteristics of the HCW, the working devices and procedures, the working environment and conditions influence trends in occupational injuries [6, 9]. The risk of transmission of bloodborne pathogens increases with their level of endemicity within the catchment area [10]. Measures for Infection prevention and control in healthcare settings include the observance of Standard Precautions, vaccination and enforcement of hospital hygiene practices. Vaccination is one of the most efficient intervention for infection prevention. Elsewhere, it is mandatory for pertussis, tuberculosis, measles, mumps, rubella, seasonal influenza, varicella and COVID-19 [6, 9, 11]. Such measures aim to protect healthcare providers, patients and visitors [9]. Additional measures such as the prohibition of needle recapping, immediately disposal of vulnerable objects in breakage-proof container and use of personal protective equipment (PPE) [12, 13]. The surveillance of AEB, their voluntary reporting and investigation are key elements of a quality healthcare delivery system [6, 14, 15]. The objective of this study was to assess the level of exposure to biological fluids among the HCWs and their management within a Regional level Hospital (RH).

## Methods

### Study design and period

A descriptive cross-sectional study was carried out from November 1st, 2020 to May 31st, 2021.

### Study settings

The present investigation was carried out in the Bertoua Regional Hospital (RH). Regional Hospitals are the second-level reference health facility after District Hospitals in the Cameroon healthcare pyramid. The Bertoua RH is located in the Eastern Region of the country. It covers a population of nearly 250 000 inhabitants, employs 224 people including administrative staff and has 217 beds. Annually, the Bertoua Regional Hospital offers some 16 754 consultations with 4 780 admissions [16, 17]. The study included clinical units among which the surgery, internal medicine, obstetrics & gynecology, laboratory, pediatrics, emergency and hygiene departments.

### Inclusion criteria

All HCWs the Bertoua RH involved in healthcare activities of who gave their written informed consent to participate in this study were included.

### Exclusion criteria

All HCWs who returned incomplete questionnaires or withdrew their informed consents were excluded from the study.

### Sampling and sample size

The sample size was calculated using the single proportion formula (n = [z_α/2_]^2^ *[p (1-p)]/d^2^), where z_α/2_ = 1.96 and *p* = 37% was obtained from a related study in Yaoundé [15], a standard error of d = 5% and 10% dropout rate, we obtained a sample size of *n* = 198 after applying a correction formula [18]. As the number of HCW almost equaled the minimum sample size, an exhaustive sampling method was adopted. In each clinical department, all eligible participants were enrolled.

### Data collection

The questionnaire was pre-tested at a nearby district hospital to ensure that all questions were valid and reliable and that any misunderstandings were addressed before final administration. Data addressing topics related to knowledge, experiences, reporting, management of occupational exposures to body fluids and hepatitis B vaccination status were collected using a self-administered questionnaire (Supplementary file 1).

### Data processing and analysis

Data were cross-checked, entered, recoded as necessary and analysed using R Statistics Version 4.3.1. Simple and multiple binary logistic regressions were used to assess the strength of the association between variables. The choice of predictors that best fit the model was done step by step using the Akaike Information Criterion (AIC). The model with the lowest index was selected. A *p*-value < 0.05 was considered statistically significant. Confidence intervals were estimated at 95% level of confidence.

### Variables

The dependent variable assessed the reported occurrence of occupational exposure during the last 12 months of service. Independent variables included sociodemographic characteristics (age, sex, unit, years of experience, and professional status), HCWs’ practices related to AEB prevention and compliance with standard precautions (hepatitis B vaccination, recapping of used needles ant the use of safety box). In the log-linear regression analysis, a participant was fully vaccinated upon receiving two doses of the hepatitis B vaccine.

### Ethical approval statement

The protocol was approved by Institutional Review Board (IRB) of the Faculty of Medicine and Biomedical Sciences of Yaoundé and the ethical clearance: N°212/UY1/FMS/VDRC/DAASR/CSD issued. Informed consent was obtained from participants prior to inclusion in the study. All methods were performed in accordance with declaration of Helsinki.

## Results

Out of the 200 contacted HCWs, two-third (*n* = 120) accepted to participate in this study (60%).

### Socio- professional characteristics of participants

Most participants were aged under 35 years (*n =* 67, 56%). Female participants represented 72% of the study population (*n =* 86). Nurses (*n =* 64, 53%) and surgical unit staff (*n =* 56, 47%) were the most represented. Participants under 5 years of professional experience made up 75% of the HCWs population (*n =* 90) (Table 1).

### Knowledge and practices related to AEB among HCW

**Fig. 1:**
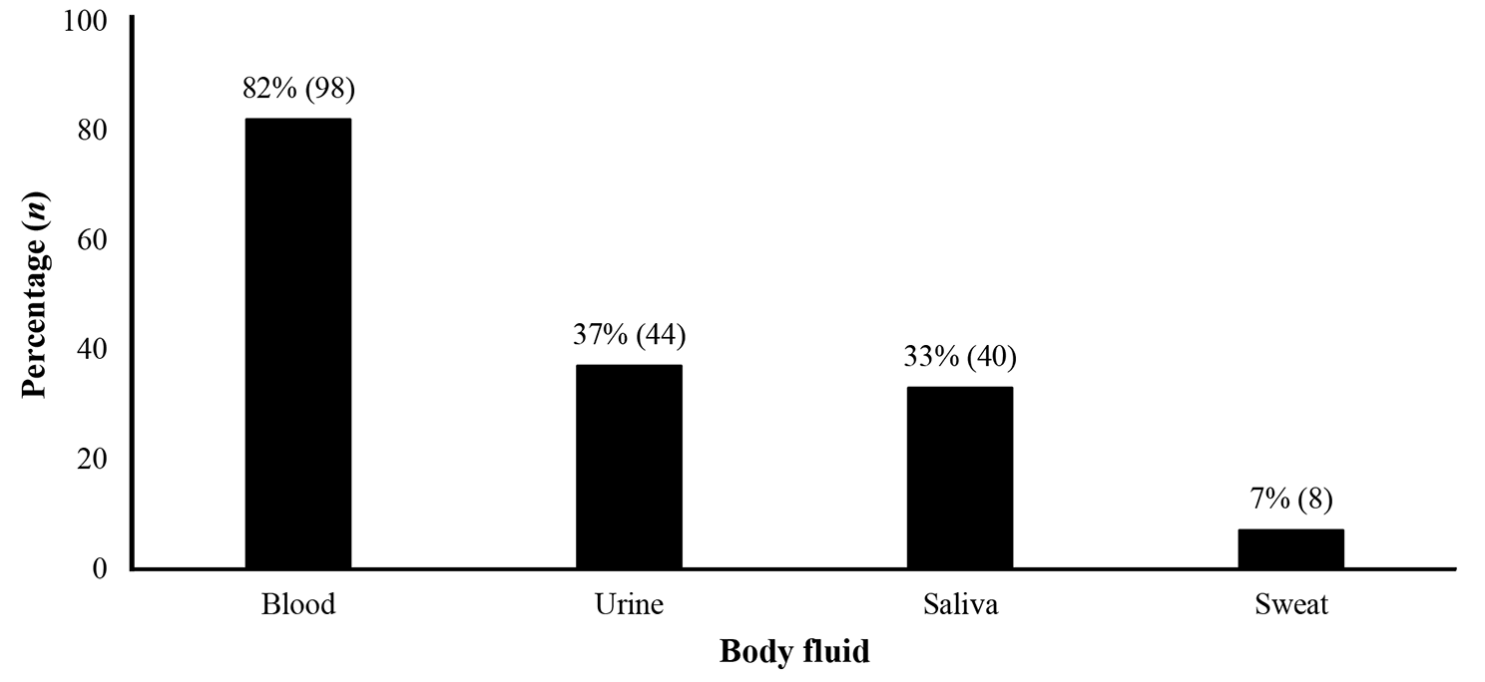
Body fluids involved in occupational exposure among healthcare workers (*n =* 120)

Participants indicated blood, urine, and saliva as main body fluids involved in accidental exposures (Fig. 1).

**Fig. 2:**
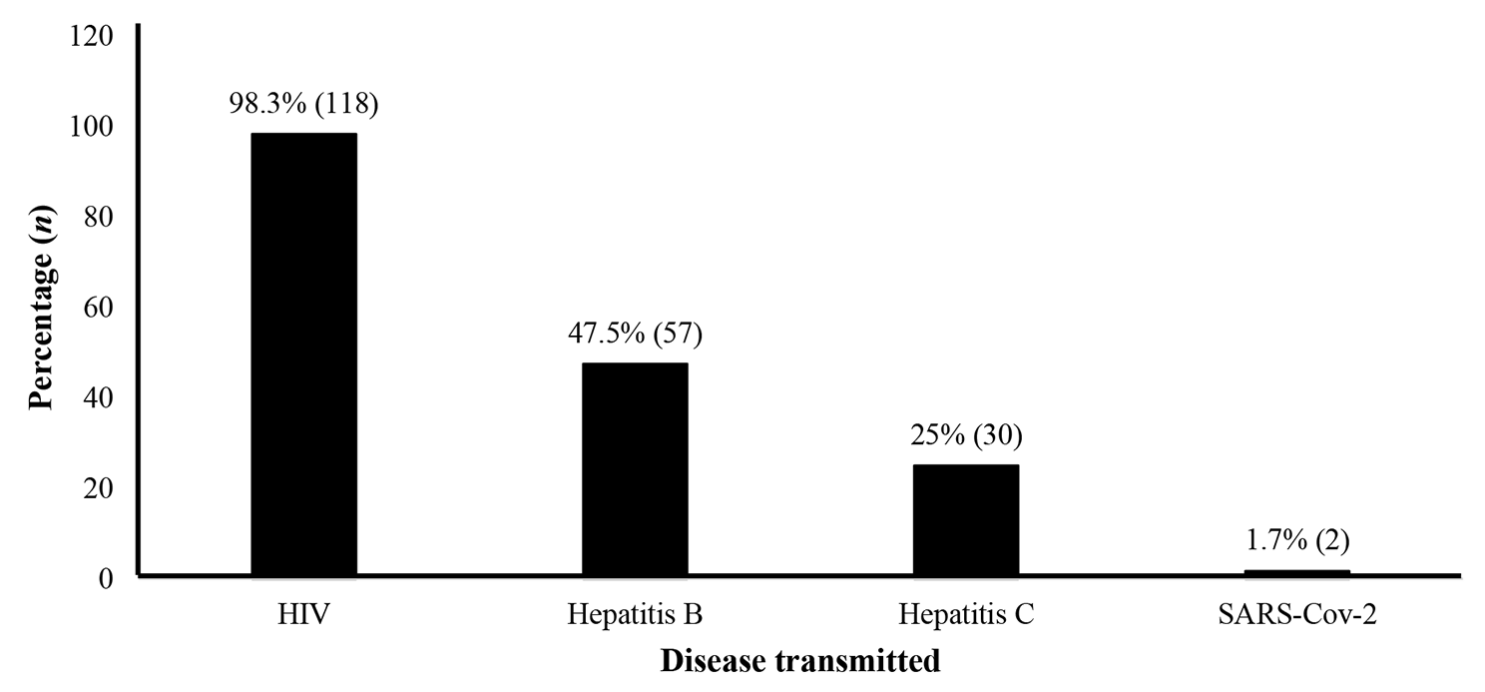
Knowledge of the diseases related to exposure to body fluids among HCWs (*n =* 120)

Almost all HCWs (> 98%) were aware that HIV/AIDS and Hepatitis B could be transmitted through AEB (Fig. 2).

### Underlying cause of AEB occurrence

**Fig. 3:**
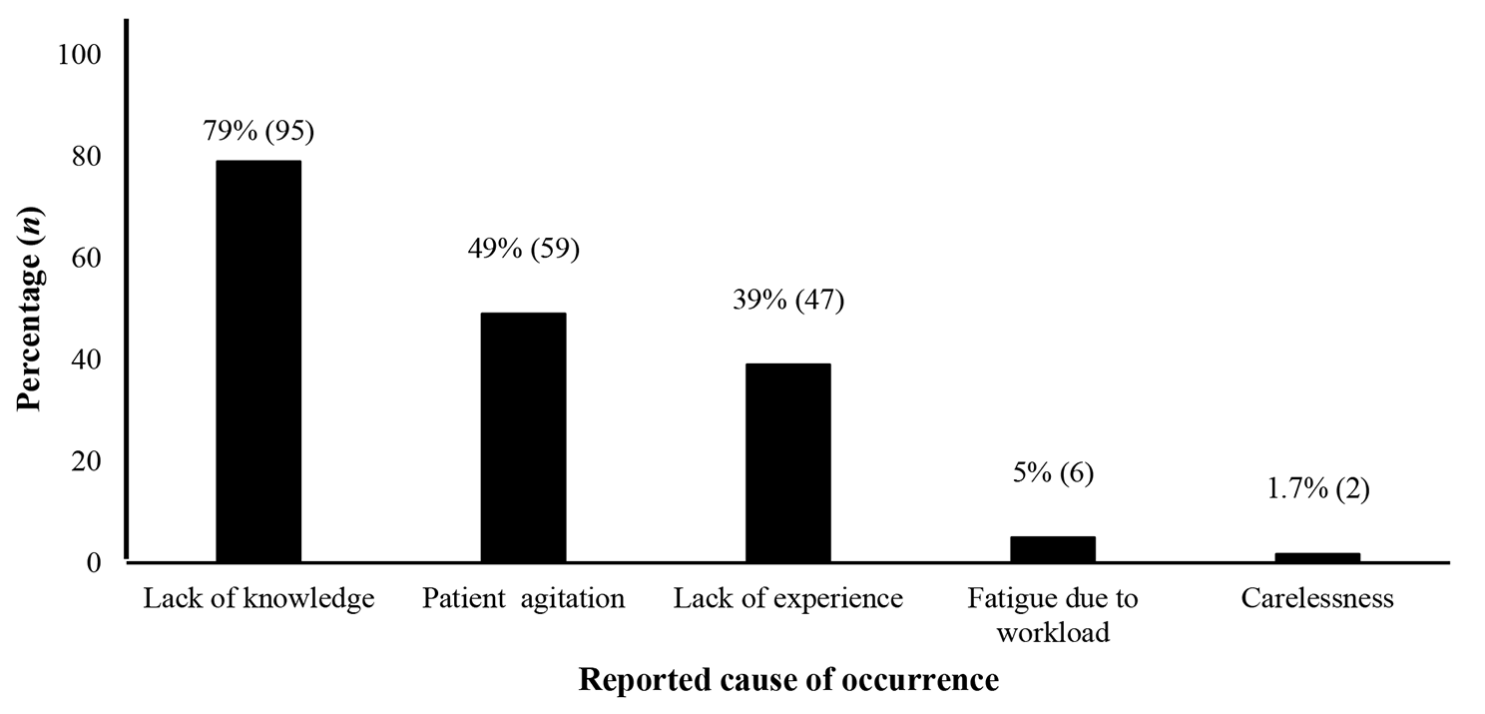
Reported factors favoring exposure to body fluids among healthcare workers (*n =* 120)

The underlying reported causes for AEB occurrence was lack of knowledge (79%) and patient agitation (49%) during care (Fig. 3).

### Training on infection Prevention in Healthcare Settings

Most HCWs had not received any training on AEB in the past three years. Less than 10% had attended an infection prevention workshop within the study year (Table 1).

### Experiences and reporting of Occupational exposures

Most HCWs had experienced at least one AEB in the last 12 months (86.7%). Out of 104 HCWs who reported to have experienced an AEB almost two-thirds did not report the incident (62%). The existence of a post exposure management facility was known to more than half of respondents (55%).

**Fig. 4:**
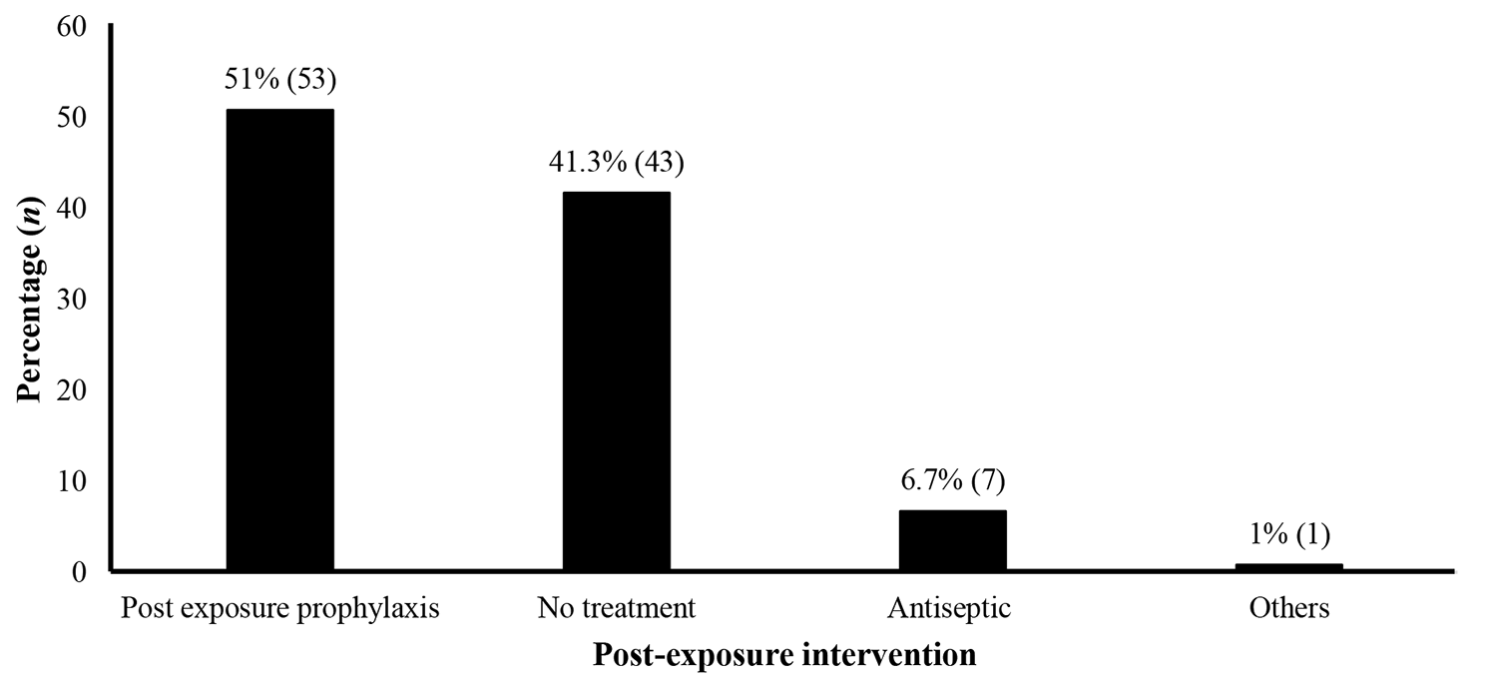
Case Management options following accidental exposure to body fluids among healthcare workers (*n =* 104)

Half of HCWs were aware of post-exposure management including the use of anti-retroviral prophylaxis (Fig. 4).

### Use of PPE & splash prevention

**Fig. 5:**
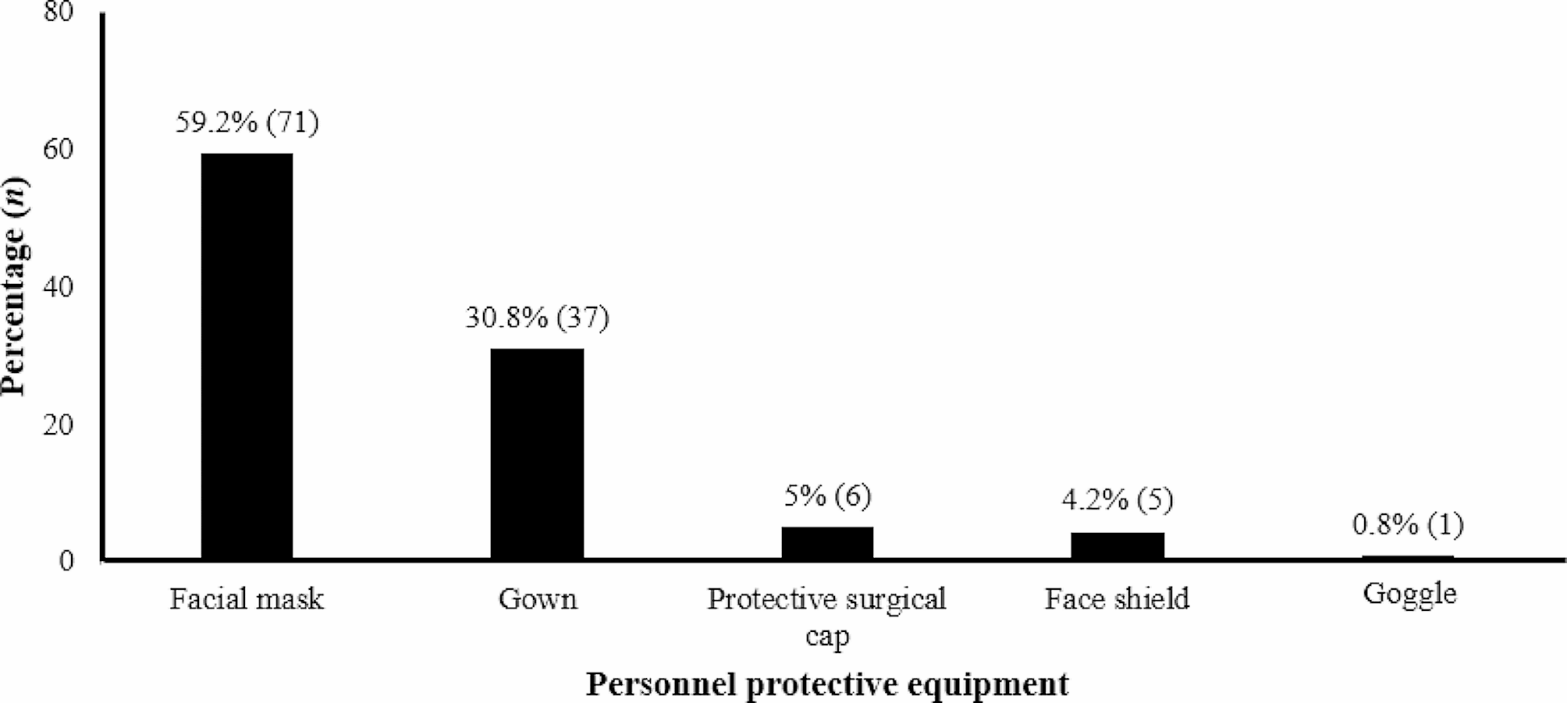
Use of Personal Protective Equipment for Splash Prevention among Healthcare Workers (*n =* 120)

Half of participants reported that they systematically used PPE. Face masks and gowns were the most mentioned (Fig. 5).

### Hepatitis B vaccination

Less than 5% (4.2%) of HCWs were fully vaccinated against hepatitis and less than a third had received an incomplete course of the vaccination (18%). Most of the unvaccinated HCWs (84.9%) had experienced exposure over the year (Table 1).

### Factors associated with AEB

Male HCWs were 5.64 times more likely to experience an occupational exposure. However, the difference was not statistically significant (CI:0.74–131; *p*-value = 0.1583).

HCWs aged over 35 years were 4.19 more likely to experience AEB. The difference was however not statistically significant (*p-*value = 0.0790).

Midwives/Nurses (aOR = 65.9; *p*-value = 0.0005) and cleaners (aOR = 14.7; *p*-value = 0.0438) were at highest risk of occupational exposure than general practitioners (Table 1).

**Table 1:** Simple and multiple binary logistic regression parameters for occupational exposure to body fluids among healthcare workers (*n =* 120)

Risk Factor	Occupational Exposure n (%)		Total (100%)	cOR	*p*-value	aOR	95% CI		*p*-value
	Yes *n* = 104	No *n* = 16					Lower	Upper	
**Sex**									
Female	72 (83.7)	14 (16.3)	86	1		1	—	—	
Male	32 (94.1)	2 (5.9)	34	3.11	0.1483	5.64	0.74	131	0.1583
**Age group (years)**									
<35	56 (83.6)	11 (16.4)	67	1		1	—	—	
35+	48 (90.6)	5 (9.4)	53	1.89	0.2692	4.19	0.95	25.4	0.0790
**Unit/Service**									
Non-clinical	22 (75.9)	7 (24.1)	29	1					
Obstetrical	19 (86.4)	3 (13.6)	22	2.02	0.3552				
Surgery	50 (89.3)	6 (10.7)	56	2.65	0.1113				
Medical	13 (100.0)	0 (0.0)	13	-	-				
**Professional experience (years)**									
<5	76 (84.4)	14 (15.6)	90	1					
5+	28 (93.3)	2 (6.7)	30	2.58	0.2290				
**Professional status**									
Generalist/Specialist	5 (50.0)	5 (50.0)	10	1		1	—	—	
Midwife/Nurse	4 (4.9)	77 (95.1)	81	19.2	**0.0003**	65.9	8.49	1428	**0.0005**
Laboratory	5 (45.5)	6 (54.5)	11	1.20	0.8351	2.60	0.20	68	0.4860
Cleaner	2 (11.1)	16 (88.9)	18	8.00	**0.0340**	14.7	1.44	386	**0.0438**
**Last training on ICP (years)**									
1–3	30 (83.3)	6 (16.7)	36	1					
3+	74 (88.1)	10 (11.9)	84	1.48	0.4838				
**Hepatitis B vaccination**									
No	79 (84.9)	14 (15.1)	93	1					
Yes	25 (92.6)	2 (7.4)	27	2.22	0.3140				
**Needle recapping**									
One handed	18 (94.7)	1 (5.3)	19	1					
Two handed	86 (85.1)	15 (14.9)	101	0.32	0.2826				
**Use of safety box**									
Yes	44 (84.6)	8 (15.4)	52	1					
No	60 (88.2)	8 (11.8)	68	1.36	0.5642				

*cOR: Crude Odds Ratio; aOR: Adjusted Odds Ratio; CI: Confidence Interval; ICP: Infection Control and Prevention*

## Discussion

Most HCWs had experienced AEB (86.7%) over the last year, but nearly two-thirds of incidents (62%) went unreported. The predominance of the female (72%) gender could be related to the growing feminization of the nursing profession in Cameroon [6, 15] and Africa [19, 20]. HCW injuries were related to host and institutional factors, inclusive of fatigue and stress related to long working hours.

### Knowledge and practices related to infection control

HCWs were aware that blood-borne pathogens (HIV, Hepatitis B and C) could be transmitted through AEB. The priority identification of HIV (98.3%) would be due to the fact that this blood-borne virus and its derivatives are well known because of their global health consequences. Such focus is valid for other HIV high risk foci including Sub-Saharan Africa [20–22]. Factors that increase the transmission of the aforementioned infectious agent from patient to HCW following a needle stick or sharps injury include a deep wound, visible blood on the device, a hollow-bore needle filled with blood, use of the contaminated device to access an artery or vein, and a high viral load status of the patient [22].

The fact that more than half of HCWs (84/120) had not attended any training on occupational exposure to body fluids in the last three years is a major preoccupation. Training is a major contributor to the observance of standard precautions and other skills. There is a need for the implementation of infection prevention and control interventions in healthcare settings. Such interventions should target HCW, visitors, patients and their caregivers. Occupational exposures are not well documented in health facilities. Similarly, post-exposure management guidelines are poorly implemented. Short-term training options, including seminars and workshops on hospital infection control are warranted [9, 12].

Both HCW and patient related factors were associated with AEB. Our study retrieved some patient factors like agitation during care, jerky movement during blood sampling, administration of injections… All interventions focusing on the use of non-threatening body language, positive tone of voice, gestures and facial expressions reduce patient’s agitation and jerky movements and could decrease AEB occurrence [23].

Factors related to HCW included poor awareness or knowledge, insufficient training and insufficient health promotion activities, fatigue and stress related to long working hours. A study identified fatigue (5%) to be a contributing factor in medical errors and near misses. Fatigue is associated with reduced cognitive performance, decreased attention and vigilance. Fatigue has been associated to poor nursing performance and increased exposure to body fluids and needle stick injuries [24]. These observations corroborate findings in Cameroon and Ethiopia [12, 25]. Similarly, poor patient cooperation during healthcare has been associated to accidental exposure to body fluids [26, 27].

Risk factors associated with needlestick and sharp injuries exposure include overuse of injections and unnecessary sharps, lack of supplies (disposable syringes, safer needle devices and sharps disposal containers), lack of access to and failure to use sharps containers immediately after injection, inadequate or short staffing, recapping of needles after use, lack of engineering controls such as safer needle devices, passing instruments from hand to hand in the operating room, lack of awareness of the hazard and lack of training [18, 22].

Universal precautions are administrative measures that entail the implementation of practices, the supply of equipment to protect HCW where exposure to blood or body fluids is likely to occur [9, 22]. While most HCWs reported using PPE systematically, a few were non-compliant, mentioning obstacles to the use of face mask and gowns. Despite the increased interest in hospital infection control with the advent of the COVID-19 pandemic, failures in the PPE supply chain were reported in several resource limited settings [21, 28, 29].

### Experience and reporting patterns of AEB

Even though more most of the HCWs had experienced AEB (86.7%), most victims did not report the incident (62%). This could be explained by the low infectious risk perception [6, 15, 21]. After an exposure to body fluids, it is recommeded that the site should be washed with soap and water; mucous membranes should be flushed with water [6, 9]. Failure to report occupational exposure may compromise appropriated post-exposure management including post-exposure prophylaxis for HIV and hepatitis virus and assessment of occupation hazards and preventive interventions [22]. Healthcare institutions should assess specific reasons for under-reporting and eliminated barriers to effective exposure control-program [21].

The risk of exposure to body fluids increased with age and professional experience. Older HCW develop more confidence and adhere less to standard precautions. Such observations corroborate reports in district level health facilities in Yaoundé [6].

There is a need for yearly refresher targeted trainings for HCWs. Such trainings reinforce good practice. Ideally, a hospital infection control committee is set up to regularly review and analyze data from the institution’s experience with exposures and near misses to determine the need for change.

The fact that half of the injured HCW (50.8%) reported that they received a course of post-exposure antiretroviral therapy indicates their awareness of the risk of contamination. The action taken by hospital managers in availing antiretroviral drugs indicates their availability to address issues related to HCWs’ exposure. There is a need to take advantage of this risk perception to sustain the implementation and scale up infection prevention and control activities [15, 21, 30].

Unvaccinated HCWs (84.9%) experienced at least one AEB in the last 12 months, denoting their vulnerability. This situation is critical, considering the high seroprevalence of HBV (11.2%) in Cameroon [31]. The main reported reasons for non-compliance with Hepatitis B vaccination were its cost, lack of time and fear of adverse events [6, 15]. Lack of time as a correlate of long working hours are mediators of work-related stress, which is associated with medical errors, poor compliance with standard practice and healthcare related infections [10, 32].

### Limitations

The main limitation of this study was the potential for recall bias regarding the different exposures experienced. In addition, during interpretation on these results, the reader should consider potential selection bias due to nonresponse. Further, the sample size used may not have been large enough to detect the significance of differences for some variables that were reported as not statistically significant. This could have narrowed the confidence intervals, bringing more precision to the measurements. However, the present study provides an insight of the risk of infection related to exposure to splashes and NSI. Further studies of microbial carriage on hands, environmental surfaces would provide further evidences for the design of IPC interventions.

## Conclusion

Over 86% of healthcare workers reported at least one accidental exposure to body fluids in the last 12 months. Midwives and nurses were disproportionally affected socio-professional groups. Two third of the AEB were undeclared. There were major insufficiencies in the observance of standard precautions, including low coverage in hepatitis B vaccination. There is a need to strengthen the implementation of comprehensive infection prevention activities in health facilities. Measures should be implemented for the systematic reporting and management of AEB in healthcare settings.

### Electronic supplementary material

Below is the link to the electronic supplementary material.

Supplementary Material 1

## Data Availability

All data generated or analyzed during this study are included in this published article.

## Abbreviations

AEB: Accidental Exposure to Body fluids
COVID-19: New Coronavirus 2
HBV: Hepatitis B Virus
HCV: Hepatitis C Virus
HCW: Healthcare Worker
HIV/AIDS: Human Immunodeficiency Virus/Acquired Immunodeficiency Syndrome
PPE: Personal Protective Equipment
RH: Regional Level Hospital
