# Supplementary material for: Awareness of standard precautions, circumstances of occurrence and management of occupational exposures to body fluids among healthcare workers in a regional level referral hospital (Bertoua, Cameroon)
Source: BMC Health Serv Res. 2024 Apr 3;24:424. doi: 10.1186/s12913-024-10855-x (PMC10993453; doi:10.1186/s12913-024-10855-x)
Supplement: Supplementary file 1 — Supplementary Material 1 [file 12913_2024_10855_MOESM1_ESM.docx]

**QUESTIONNAIRE**

| Socio-demographic profile | |
| --- | --- |
| Q01. Age ........... | /___/ |
| Q02. Sex: 1- Female; 2- Male | /___/ |
| Q03. Socio-professional category: 1- Specialist doctor; 2- Dental practitioner; 3- General practitioner; 4- State registered nurse; 5-Midwife 6- Nursing assistant; 7- Auxiliary service agent (stretcher-bearers); 8- Laboratory technician 9-Cleaner | /___/ |
| Q04. Department: 1- Internal medicine ; 2- Surgery ; 3- Paediatrics ; 4- Gynaecology and obstetrics ; 5- Emergency ; 6- Hygiene; 7.Laboratory | /___/ |
| Q05. Year of experience :……………………………… |  |
| General knowledge and attitude |  |
| Q06. What is a occupational exposure to body fluids?  1. Involuntary contact with blood and/or body fluids during a medical procedure;  2. Contact with blood during surgery in a hospital ward;  3. Voluntary contact with blood during a medical procedure;  4. Don't know | /___/ |
| Q07. What are the biological fluids involved in occupational exposures?  1-Blood; 2- Saliva; 3- Urine; 4- Sweat; 5- Semen; 6-All the answers are correct; 7- Others(specify) :................................ | /___/ |
| Q08. Have you received training in the prevention of illnesses related to accidents involving exposure to biological fluids in hospitals?  1- Yes ; 2- No ; | /___/ |
| Q09 If yes, when did you attend?   1. less than a year ago; 2. between 1-3 years ago; 3. more than 3 years ago | /___/ |
| Q10. Which of the following diseases can be transmitted through exposure to blood and/or other body fluids?  1- Viral hepatitis B; 2- Viral hepatitis C; 3- HIV/AIDS; 4- Corona virus; 5- Other (specify):............................... | /___/ |
| Q11. What are the causes of accidents involving exposure to blood and other biological fluids?  1- Lack of experience; 2- Lack of knowledge; 3- Negligence ;  4- Work-related fatigue; 5- Poor quality of equipment  5- Other (please specify):........................................................... | /___/ |
| Q12. Does the hospital have a system to manage occupational exposure to blood and/or other biological fluids?  1- Yes ; 2- I don't know | /___/ |
| Q13. Do you systematically used Personal Protective Equipment to prevent splash exposure?  1-Yes ; 2- No | /___/ |
| Q14. Have you heard of prophylaxis after accidents involving exposure to blood and/or other biological fluids?  1- Yes ; 2-No | /___/ |
| Q15: What is the prophylaxis used after accidents involving exposure to blood and/or other biological fluids?  1- Administration of drugs to treat HIV  2- Treatment to prevent contamination with HIV, viral hepatitis B after exposure to blood and/or other biological fluids  3- Quarantine after exposure to blood and/or other biological fluids  4- Other (please specify):........................................................... | /___/ |
| Q16 What medication is used after accidents involving exposure to blood and/or other biological fluids?  1- Antibiotics (penicillin)  2- Anti-retroviral prophylaxis  3- Antiseptics  4- Other (please specify): ........................................................... | /___/ |
| Experience of Occupational exposure to body fluids |  |
| Q17. Have you had an accident involving exposure to blood and/or other biological fluids in the last 12 months?  1-Yes ; 2- No | /___/ |
| Q18. Did you report the accident to the organization responsible for dealing with accidents involving exposure to blood and/or other contaminated biological fluids?  Yes ; 2- No | /___/ |
| Q19. If yes, did you receive prophylaxis after exposure to blood and/or other contaminated biological fluids?  Yes ; 2- No | /___/ |
| Q20. What prophylactic medication had you received?  1- Antibiotics (penicillins)  2- Anti-retroviral agents  3- Antiseptics  Other (please specify): ......................................................... | /___/ |
| Q21. What protection methods usually do you use to prevent needlestick injuries?  1- One-handed recapping; 2- Two-handed recapping  3- Other (please specify):............................................................. | /___/ |
| Q22. What methods of protection against splashes do you usually use?  1- Wear facial mask ; 2- Wear gown ; 3- Wear gloogle ; 4- Wear face shield ; 5- Wear a surgical protective cap  6- Other (please specify):............................................................. | /___/ |
| Q23. What container do you usually used for needle and sharp objects disposal?   1. Safety box; 2- Plastic bottle ; 3- Carton container 2. Other(please specify)___________________________________________ | /___/ |
| Q24. Are you vaccinated against viral hepatitis B? | /___/ |
| Q25. How many doses of vaccine have you received? ……………………… |  |
